# Clinical and hematological findings in alpacas (*Vicugna pacos*) with and without *Candidatus* Mycoplasma haemolamae infection

**DOI:** 10.1038/s41598-024-70956-9

**Published:** 2024-08-30

**Authors:** Matthias Gerhard Wagener, Saskia Neubert, Frederik Kiene, Johannes Buchallik-Schregel, Thies J. Nicolaisen, Benjamin U. Bauer, Alexandra von Altrock, Thekla Großmann, Antje Polifka, Martin Ganter

**Affiliations:** Clinic for Swine and Small Ruminants, Forensic Medicine and Ambulatory Service, University of Veterinary Medicine Hannover, Foundation, Hannover, Germany

**Keywords:** Anemia, South American camelids, Hemotropic mycoplasma, Blood smear, Clinical findings, Clinical microbiology, Animal physiology

## Abstract

Anemia is a common problem in South American camelids (SACs). Infections with *Candidatus* Mycoplasma haemolamae (*CMh*), a cell-wall free, hemotropic bacterium, are often suspected to be an important cause of anemia, as the pathogen infects the erythrocytes and is found in the blood of up to 30% of SACs. The information on the clinical signs of animals infected with this pathogen vary widely. Most infections are clinically inapparent. Treatment is usually carried out with oxytetracycline. A detailed overview of the clinical and hematological findings in 13 alpacas infected with *Candidatus* M. haemolamae (CMh+), based on patients from our university clinic and comparing those findings with the results of 22 negative alpacas (CMh−) is provided. Assignment to both groups was based on the PCR result. No relevant clinical or hematological differences between CMh+ and CMh− were found, the clinical signs in CMh+ were usually due to comorbidities. The examination of a blood smear alone proved to be insufficient; a PCR test should be carried out to confirm or rule out an infection. A critical review of the need for antibiotic treatment on the basis of a positive test result alone is recommended.

## Introduction

South American camelids (SACs: alpacas and llamas), originally native to the Andes, have become an established livestock in Europe^1^. This has resulted in an increasing number of alpacas being presented for veterinary care in central Europe^2^. Common health problems in alpacas include gastrointestinal disorders, with endoparasites playing a major role; skin problems, often due to mange; colic, which can be caused by ulceration of the compartments; or tooth root abscesses^3^. Many of the alpacas presented to the veterinary clinic reveal anemia, which can develop into a life-threatening condition for the animal^4,5^. Severe anemia in SACs is often caused by *Haemonchus contortus*, which can lead to massive blood loss^6^. Another pathogen associated with anemia in alpacas and llamas is *Candidatus* Mycoplasma haemolamae (*CMh*)^3,7^. Formerly known as Eperythrozoon, the pathogen is a wall-less, hemotropic bacterium that is regularly detected in the blood of alpacas or llamas^8^. Transmission of hemotropic mycoplasma can usually occur through any transfer of infected red blood cells from one animal to another, for example, during vaccination or shearing^7^. There are hints for vertical transmission of *CMh*^9,10^, but it remains unknown whether the pathogen is transmitted in utero or during parturition^9^. However, transmission via colostrum seems to be unlikely^11^.

An early description of the pathogen was made by McLauglin et al. in 1990 after identification of hemotropic bacteria in blood smears of llamas^12^. With the possibility of detection by polymerase chain reaction (PCR)^13^, data on the epidemiology of the pathogen from different countries have been published. In llamas and alpacas from Peru, the prevalence was 15.8% and 19.3%, respectively, whereas alpacas from Chile had a lower prevalence of 9.3%^14^. Studies in South American camelids that are kept in Europe showed similar prevalences; PCR testing of 225 alpacas and llamas from Germany and Switzerland revealed that 18.7% of the samples were positive for *CMh*^15^. Another study from Switzerland showed a prevalence of 18.6%, with 39.1% of the farms tested having positive animals^16^. In SACs from Austria a significant difference in the prevalence of llamas (17.6%) and alpacas (31.7%) was found by Franz et al.^17^. Crosse and others reported a prevalence of 29.0% in an alpaca herd of 131 animals in the UK^18^. However, a study of alpacas in New Zealand reported a significantly lower prevalence, with only two of 206 alpacas tested revealing a positive PCR result (0.97%)^19^. More recent first descriptions of *CMh* in SACs from Italy with 14 positive of 20 animals (66.7%)^20^ or Finland (35.3% positive animals)^21^ were published in 2022.

Most infections with *CMh* have been described to be clinically inapparent^3,14,22^, but also severe courses have been documented so far^12,23,24^. Clinical symptoms usually occur in association with stressors such as other underlying diseases, or shipping^18,25^.

Clinical findings observed in South American camelids associated with *CMh* infections seem to be non-specific. Among others, lethargy, tachycardia, tachypnoea, acidosis, azotemia, poor body condition due to chronic weight loss, pale mucous membranes, and also death have been described so far^10,12,18,25^. The infection has also been associated with abortions, spiral colon impaction, or severe pulmonary edema^26–28^. Icterus, as described in infections with hemotropic mycoplasmas of other species, is not usually observed in *CMh* infections^7,12^. Antibiotic treatment with oxytetracycline is generally used as therapy; however, not every infection with *CMh* is cleared consistently^18,25^.

Pathological findings associated with an infection with *CMh* were mycotic abomasitis, encephalomyelitis, fibrinous polyserositis, hemorrhagic enteritis, septicemia, polyserositis, interstitial pneumonia, splenic hyperplasia, or hydrothorax^12,29^. Nonetheless, *CMh* does not appear to play a major role in the total number of South American camelids that underwent necropsy. In the retrospective evaluation of 6757 laboratory submissions of South American camelids from England and Wales, the pathogen was only detected six times^30^. However, it can be assumed that not every submission was tested for the pathogen in that study^30^. In other retrospective evaluations of pathological data from South American camelids, *CMh* is also mentioned only occasionally or not at all^31,32^.

Nevertheless, there are only few combined clinical and laboratory diagnostic data from clinically abnormal animals available in which infection with *CMh* has been detected.

The aim of this study was to provide an overview of the clinical and hematological findings of hospitalized alpacas with *CMh* infection that was confirmed by PCR. Moreover, the findings from these animals with a group of alpacas that revealed a negative PCR result to identify existing differences of infected and non-infected animals were compared.

## Materials and methods

### Data collection

For the retrospective data analysis, medical records from the Clinic for Swine, Small Ruminants, Forensic Medicine and Ambulatory Service of the University of Veterinary Medicine Hannover, Foundation, Hannover, Germany were screened for SACs that had undergone PCR testing for *CMh* in the period January 2021 to January 2024. This was performed by real-time-PCR from EDTA-blood, which was sent to external diagnostic laboratories (SYNLAB.vet GmbH, Augsburg, Germany or LABOKLIN, Bad Kissingen, Germany). Both laboratories were accredited in accordance with DIN EN ISO 17025:2018.

In addition, complete information on sex (female, male, male-neutered) and age (in days as difference between date of birth and date of sampling) of the animal, length of hospital stay (in days) as well as outcome (released, deceased, euthanized) from each animal was recorded. If euthanasia was performed, it was carried out by intravenous administration (*V. jugularis*) of pentobarbital (83–122 mg/kg bodyweight Euthadorm® 500 mg/mL CP-Pharma Handelsgesellschaft mbH, Burgdorf, Germany). Further inclusion criteria was a hematological examination from an EDTA-blood sample taken from a jugular vein of the animal (EDTA Monovette 9 mL K3E, Sarstedt AG & Co. KG, Nümbrecht, Germany)^33^. This included the following parameters: white blood cell (WBC) count [×10^9^/L], which was determined microscopically in a Neubauer counting chamber after 5 min lysis of 100 µL EDTA blood in 900 µL 3% acetic acid solution^33^; packed cell volume (PCV) [L/L], which was determined after centrifugation of EDTA-blood in a microhematocrit tube for 10 min at 10,000×*g*^33^; hemoglobin (Hb) [g/L], which was determined photometrically using a cyan solution; mean cellular hemoglobin concentration (MCHC) [g/L]^33^, which was calculated according to the following formula: MCHC [g/L] = Hb [g/L]/PCV [L/L]; as well as microscopical differentiation of WBC in a blood smear stained according to Pappenheim^34^ at 1000 times magnification (lymphocytes, segmented neutrophils, band neutrophils, eosinophils, basophils, metamyelocytes, myelocytes, monocytes each [%] and then calculated with the WBC in [×10^9^/L])^33^. Normoblasts (nucleated red blood cells) were additionally recorded [1/100 WBC]. Reticulocytes were not routinely examined, so data were not available for all hematological findings. Reticulocyte counts were determined microscopically by counting the proportion of reticulocytes per 1000 erythrocytes (RBC) in blood smears stained with brilliant cresyl blue [1/1000 RBC] at 1000 times magnification^33^.

The exact methods for hematological examination have been described previously^4,33^. Interpreting the hematological results was performed according to the reference intervals for alpacas from Dawson et al.^35^. According to Dawson et al. a PCV below 0.22 L/L was used as the definition for anemia. Further classification of the severity of anemia was made according to Franz and Wittek^36^. The erythrocytes in the monolayer of the blood smears were checked microscopically for anisocytosis, poikilocytosis and polychromasia at 1000 times magnification. The extent of the changes were scored from 0 to 4 (0: the change does not appear microscopically in any field of view; 1: the change appears only occasionally not in every field of view; 2: the change appears in one to three RBC in every field of view; 3: the change appears in more than three RBC in every field of view; 4: the change appears in more than 50% of RBC). In addition, the presence of basophilic dots (as a hint for mycoplasmas), Howell-Jolly bodies, and Cabot rings were noted [yes/no]. Abnormal erythrocyte shapes were recorded as additional text.

Clinical parameters (body weight [kg]; rectal temperature [°C]; respiratory rate [1/min]; heart rate [1/min]; Body Condition Score (BCS) and FAMACHA©-score) were assessed as part of the routine protocol of the clinic and interpreted according to Whitehead^3^ and Wagener and others^37,38^. Fecal samples were analyzed according to the routine protocol of the clinic^39^. This method was adapted to the extent that a minimum of 10 g of feces was measured accurately and used to calculate the number of eggs shed per gram of feces. In contrast to the McMaster technique, this process combines the Baermann-Wetzel larval migration method with two combined sedimentation-flotation approaches. After sedimentation with distilled water, in the first approach saturated saline solution is applied, mainly for the detection of strongly type eggs; in the second approach sodium silicate solution is used, mainly for the detection of liver fluke eggs. The results were interpreted according to Neubert et al. making a subdivision into low-grade, medium-grade, or high-grade for the severity of the infestation with gastrointestinal nematodes^40^. Furthermore, the final diagnoses of the animals with some animals having multiple diagnoses were also included in the evaluation.

As only data from three llamas were available, further evaluation focused on alpacas alone. These were divided into two groups:

1. Alpacas with negative PCR-test for *Candidatus* M. haemolamae (CMh−) (n = 22)
2. Alpacas with positive PCR-test for *Candidatus* M. haemolamae (CMh+) (n = 13).

### Statistical analysis

Statistical analysis was performed with Excel (Microsoft Excel for Office 365) and SAS (SAS Enterprise Guide 7.1). Descriptive statistics included means, standard deviation (SD), median, minimum, and maximum. Numeric data were checked for normal distribution using the Shapiro–Wilk test. Groups with normally distributed data were compared using the t-test, and groups with non-normally distributed data were compared using the Mann–Whitney U test. The two-sided Fisher exact test was used to test categorical data for clustering of clinical or laboratory diagnostic findings in each group (CMh−; CMh+). A *p* < 0.05 was assumed to be significant in each case.

### Ethics statement

The study was approved by the Research Ethics Committee of the University of Veterinary Medicine Hannover, Germany under the Approval-code TiHo-REC_14_04-24, as it is compatible with the animal welfare guidelines of the University of Veterinary Medicine Hannover and with European and German animal welfare laws. Only retrospective data from animal samples taken for clinical- therapeutic reasons from patients of the Clinic for Swine, Small Ruminants, Forensic Medicine and Ambulatory Service of the University of Veterinary Medicine Hannover were used.

## Results

The relevant results are summarized in the following text. A detailed overview of the individual results in the form of descriptive statistics and pairwise comparisons can be found in Table 1. Contingency tables and Fisher’s exact test are presented in Tables 2 and 3.

### Demographic data

A total of 35 alpacas from 23 different farms were tested for *CMh* by PCR. Of these, 13 (37.1%) from 11 different farms were positive (CMh+). The CMh+ group consisted of five females and eight males (six intact, two neutered). The CMh− animals included six females and 15 males (11 intact, four neutered). Within the CMh+ group there was one male cria (< 1 year) and within the CMh− group there were three male crias. The age of the animals ranged from 43 to 4056 days (CMh+) and 206 to 5013 days (CMh−). Bodyweight ranged from 12.4 to 77.5 kg (CMh+) and 10.4 to 102.0 kg (CMh−) (Table 1).

### Clinical examination

Clinical examination revealed both hypothermia and hyperthermia in the groups. Hypothermia was detected at a higher percentage in CMh− animals, but there were no significant differences concerning rectal temperature. Tachypnoea was recorded frequently in both groups, bradypnoea in only one CMh− animal. Tachycardia and bradycardia occurred in both groups without any statistical difference (Table 2). A BCS that was too low (BCS < 2.5) was recorded more frequently in CMh− animals (*p* < 0.01). Only two CMh− animals had a BCS that was too high (BCS > 3), whereas the majority of CMh+ animals revealed a moderate body condition. The median of the BCS in CMh+ animals was higher (2.75) than in the CMh− animals (1.5), which was statistically different (*p* = 0.01) when comparing both groups. A physiological FAMACHA©-score (1 or 2) was observed in most of the animals, a fatal score of 5^41^ was present in only one CMh+ animal and three of four CMh− animals; however, this difference was not significant.

### Fecal examination

Most of the animals in both groups were infested with gastrointestinal nematodes. Endoparasite burden of the animals had different degrees (CMh+ : six animals low-degree, one animal medium-degree, and one animal high-degree; CMh−: eight animals low-degree, one animal medium-degree, four animals high-degree, three animals severe). *Eimeria macusaniensis* was not present in the fecal samples of any CMh+ animal, but in two of the CMh− animals. There was no statistical difference concerning the fecal examination.

### WBC count

The WBC count revealed leukopenia and leukocytosis in both groups, Lymphopenia and lymphocytosis also occurred sporadically in both groups. Neutropenia was seen less commonly than neutrophilia; however, this was not significant. Band neutrophils were detected in the blood smear of almost all animals. Other premature stages of neutrophils (metamyelocytes and myelocytes) were also seen in single cases in both groups. In neither group was there any animal with eosinophilia or basophilia. Nonetheless, there were some animals in which no eosinophils or basophils could be detected in the blood smear. Monocytosis was only detected in two CMh− animals. The neutrophil-to-lymphocyte ratio (NLR) was increased in a lot of animals in both groups (reference interval alpacas: 0.5–2.9^42^). To date, there are no published reference values for the lymphocyte-to-monocyte ratio (LMR) in alpacas. Nonetheless it was noticeable that the CMh+ animals had a wider range (2.29–79) than the CMh− animals (1.43–31). None of the parameters of the WBC showed a statistical difference between CMh+ and CMh− animals.

### RBC count

Eighteen of the animals revealed anemia (PCV < 0.22 L/L^35^), which was present in more than half of the CMh− animals and in about 30% of the CMh+ animals. Two of the CMh+ animals had a fatal PCV of 0.05 L/L each, five of the CMh− animals had a fatal PCV of 0.04 L/L to 0.07 L/L. However, three of four animals with a BCS of 1 also had a high gastrointestinal nematode burden. Hemoglobin showed similar trends in both groups; the mean corpuscular hemoglobin concentration (MCHC) was decreased in about 40% of the animals in both groups. One CMh− revealed an increased MCHC.

All animals in both groups revealed anisocytosis in the blood smear. Polychromasia was observed in more than half of the animals in each group. Poikilocytosis was seen more frequently in the blood smears of CMh− animals than in blood smears of CMh+ animals (*p* < 0.05). The most common alterations in erythrocyte morphology in these cases were dacrocytes and spindloid cells. The animals with the higher grades in alterations of the RBC had a PCV of < 0.1 L/L. Detailed information on the findings of the red blood cell morphology in both groups is displayed in Table 4.

**Fig. 1:**
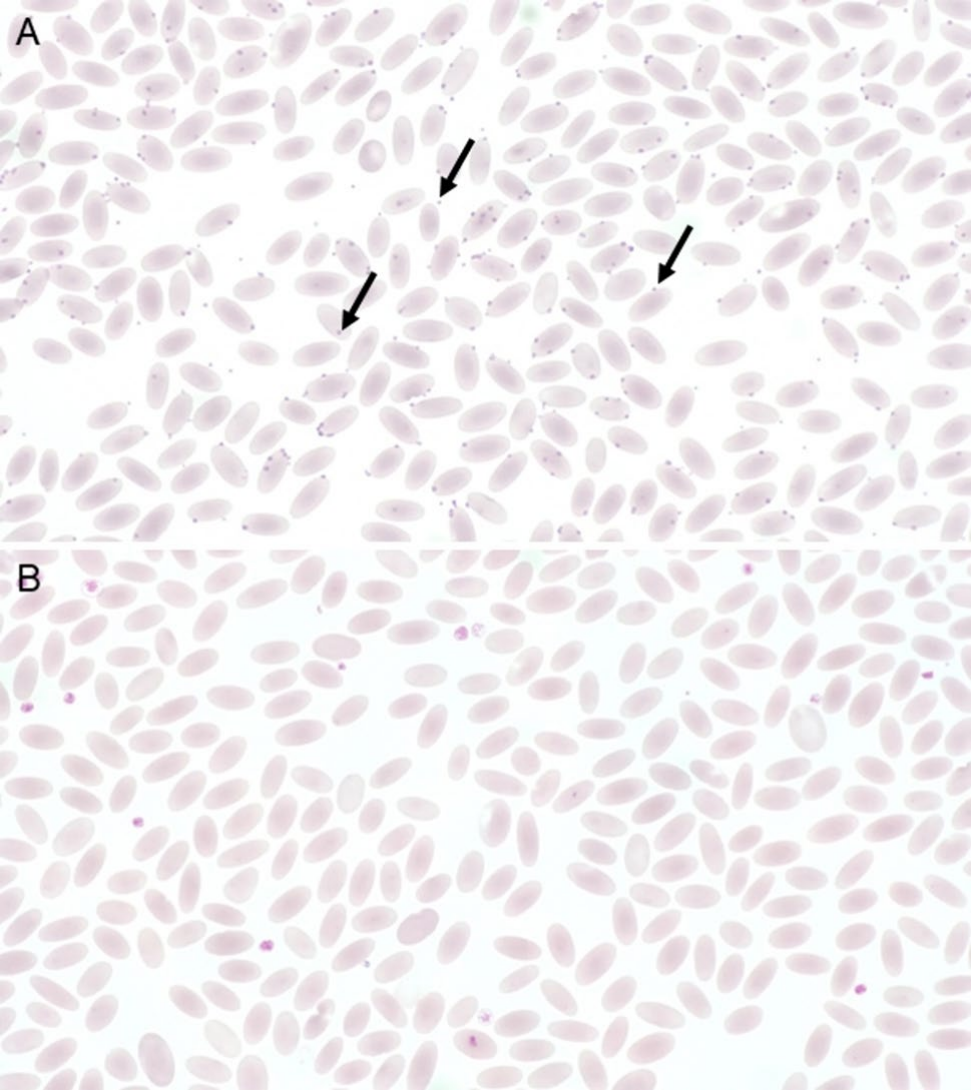
(**A**) Blood smear from a male adult alpaca with *Candidatus* Mycoplasma haemolamae. The hemotropic bacteria appear as basophilic dots (arrows). The infection was confirmed by PCR. There is mildanisocytosis. (**B**) Blood smear from the same animal after five days treatment with oxytetracycline. No pathogens are visible in the blood smear, but this blood sample still revealed a positive result for *CMh* in the PCR.

Basophilic dots (Fig. 1) in erythrocytes were detected in four blood smears of both CMh+ and CMh− animals. Howell-Jolly bodies were only seen in two blood smears of CMh− animals. Cabot rings were detected in one CMh+ and five CMh− animals.

Normoblasts were frequently present in blood smears of both groups. In almost all of the cases where normoblasts were detected the reference limit was exceeded.

Reticulocytes were determined in only a few animals. There were data for only three CMh+ animals available (0%, 0.2%, and 9.8% of the RBC, respectively). In the CMh− group, reticulocytes were detected in all 13 animals that underwent investigation for reticulocytes. In six of these animals the reticulocytes made up more than 10% of the erythrocytes. However, apart from the finding of poikilocytosis, no parameter of the red blood count was statistically different between the groups.

### Clinical diagnoses

#### *Candidatus* Mycoplasma haemolamae positive animals (CMh+)

Other diagnoses than infection with *Candidatus* M. haemolamae included hemonchosis in two animals, chorioptic mange in two animals as well as pneumonia, endometritis, colic, and lameness of an unknown cause in one animal each. In five animals, the main diagnosis was limited to infection with *CMh*. Three of these animals had been previously clinically unremarkable and presented as companion animals for another hospitalized animal from the same flock. One animal had been previously found to be weak by the owners. However, no detailed clinical examination was carried out on this animal, as only ambulatory blood samples were taken at the clinic and the animal was then returned home. The blood count of this animal did not show any deviation from the reference limits except for the presence of 0.55 G/L band neutrophils. In another animal (neutered male adult), a tremor was observed by the owners. Nevertheless, this was not observed during hospitalization of this animal. Apart from tachypnoea (48/min), no abnormalities were found on clinical examination. Laboratory diagnostics of this animal revealed increased band neutrophils (4.9 G/L) and an increased NLR (6.87). The animal was released from hospital after 5 days.

#### *Candidatus* Mycoplasma haemolamae negative animals (CMh−)

The most common diagnosis in negative animals was haemonchosis (eight animals). However, the cause of anemia could not be clearly identified in all cases, as some of the pre-treatments included deworming before presentation at the clinic. Other diagnoses were copper deficiency; selenium deficiency; infection with *E. macusaniensis*; dental problems; colic (in two animals each); pneumonia; otitis media due to sarcoptic mange; patellar luxation; and other lameness (in one animal each). One animal was a companion animal, where also gastrointestinal nematodes but no anemia was detected.

### Outcome

The majority of the animals were released from the clinic (12/13 CMh+ and 17/22 CMh−). One CMh+ animal that revealed haemonchosis was euthanized due to the condition worsening after severe anemia (PCV: 0.05 L/L). Among the CMh− animals, two were euthanized, one animal due to worsening of its condition after severe anaemia (PCV: 0.05 L/L) and one animal due to patellar luxation. Three CMh− animals died due to pneumonia, cachexia, or anaemia of unknown cause. The hospitalization stay ranged from 1 to 25 days (CMh+) and from 2 to 35 days (CMh−) with means of 14.7 ± 9.5 days (mean ± SD; CMh+) and 13.5 ± 7.1 days (mean ± SD; CMh−).

#### Repeated test for *Candidatus* Mycoplasma haemolamae

Repeated PCR testing for *CMh* during hospitalization was performed in only one animal. The two-year-old male alpaca was presented due to lameness. The blood smear showed basophilic dots in approximately 80% of the erythrocytes (Fig. 1a) and the PCR test identified *CMh*. The animal was treated systemically with oxytetracycline subcutaneously (20 mg/kg bodyweight Tetroxy® Vet 200 mg/mL; Dechra Veterinary Products Deutschland GmbH, Aulendorf, Germany) from the following day onwards. After five consecutive days of treatment another blood sample was taken. This sample revealed no microscopic evidence of mycoplasmas in the blood smear (Fig. 1b), but was still positive in the PCR. Apart from tachypnoea and lameness, the cause of which could not be determined, there were no clinical abnormalities in that animal, and it was released after nine days of hospitalization.

## Discussion

Thirteen of the 35 animals examined (37.1%) were found to be infected with *CMh*. This detection rate is similar to the prevalences in Austria or England described previously^17,18^. Differences concerning sex or age were not found in the present study, which goes in hand with the findings of Kaufmann et al.^15^. However, Crosse et al. reported more infected young than old alpacas^18^. The clinical and hematological findings of the CMh+ and CMh− groups did not differ for most parameters. Whereas most of the animals in this study were emaciated and revealed a BCS < 2.5, CMh− animals had a statistically lower BCS than CMh+ animals. Decreased body condition in connection with *CMh* has been reported before^18,25^. The higher BCS in the CMh+ animals might be explained by the fact that the *CMh* infection was also an incidental finding in clinically unremarkable companion animals that were not presented due to another underlying disease. The even worse BCS in the negative animals could be attributed to the high proportion of animals infected with gastrointestinal nematodes in this group. Nonetheless, a poor nutritional status is a common observation in animals presented to the clinic^4^. On the one hand, emaciation could be due to the chronicity of underlying diseases; on the other hand, emaciated animals could also be more susceptible to diseases^43^. When interpreting the high number of animals with tachypnoea in both groups, stress was also taken into account, as the animals were transported to the clinic before the examination.

Fever, as observed in hemotropic mycoplasmas of other species^7^, was only observed in two of the positive animals, one additionally revealed pneumonia, the other revealed no further diagnosis other than *CMh*. Some of the CMh+ animals revealed no deviations in clinical parameters, which can also be found in the present literature^9,44^. Deviations in the clinical parameters that were recorded can be attributed to the diseases diagnosed in addition to the infection with *CMh*. As described by other authors^3,14,22^, no pathognomonic clinical signs of *CMh* infection could be identified in our study. Crosse et al. supposed that only stress or another underlying condition could lead to clinically apparent infections^18^. Nonetheless, it should be borne in mind that stress and other underlying conditions can also lead to severe clinical signs.

About half of the alpacas investigated in this study (18/35) revealed anemia. However, it should be considered that a selection of the animals had already taken place in advance and PCR testing for *CMh* was only performed on suspicion of infection for this pathogen like the presence of other positive animals in the flock or an anemic condition of the animal. It is therefore not surprising that many of the animals were anemic; nevertheless, anemia is a common problem in SACs presented to the veterinary clinic^4^. Anemia in SACs is often caused by *H. contortus*^6,45^, which was the most common diagnosis (10/35) besides an infection with *CMh* in our study. The 10 animals with haemonchosis had the lowest PCV with a range from 0.05–0.15 L/L, which can be classified as severe anemia^45^. As differentiation of *H. contortus* according to Colditz et al.^46^ was only performed in the feces of two animals, the diagnosis was made on the basis of a high FEC and anemia, which is a common practice in small ruminant and camelid medicine^47–49^. Two of the animals with a PCV of 0.05 L/L revealed both haemonchosis and a positive PCR result for *CMh*. Storey et al.^48^ reported a llama with a PCV of 0.05 L/L that had haemonchosis and was positive for *CMh*. The animal was found to be much more lethargic than three other llamas with a PCV of ≤ 0.1 L/L^48^. However, the impact of the mycoplasma-infection was not evaluated in that case. The influence of the mycoplasmas on the course of the disease in the case of haemonchosis was also difficult to assess in our study population, as only two animals were affected by both. Haemonchosis was assumed to be the relevant cause of disease in these cases, which is supported by the fact that two other animals in our study with haemonchosis without *CMh* did not survive.

Nonetheless, it cannot be ruled out that mycoplasma infection could be the sole cause of severe anemia. In an evaluation of blood transfusions administered to 22 alpacas with PCVs ranging from 0.05 to 0.19 L/L, Luethy et al. stated that three of the animals were transfused due to *CMh* infection^23^. Further information on these animals, such as possible comorbidities, are not reported in their paper.

Compared to the reference intervals^35^, it was striking that many of the investigated animals had anemia of either hypochromic or normochromic character. Most of these animals showed signs of regeneration like normoblasts or polychromasia^45^. Hypochromasia is a typical finding for iron deficiency anemia^50^, which remains unclear in these animals, as plasma iron content was not measured. However, in ruminants, a slightly decreased MCHC may indicate regeneration^51^. Nevertheless, it was not possible to evaluate the erythrocyte count and other erythrocyte indices (MCV and MCH) for these animals, as no manual erythrocyte count had taken place in routine laboratory diagnostics.

Viesselmann et al. also investigated PCV, RBC count, and Hb of 114 SACs, comparing the laboratory diagnostic findings of animals with (39/114) and without (75/114) *CMh* infection as well as with the FEC of the animals^22^. They could not find any significant differences in the hematological variables between *CMh* positive and negative animals, but they found a significant decrease in PCV, RBC count, and Hb in animals with high FEC. This goes in hand with the result of our animals, where almost all hematological parameters revealed no significant difference between CMh+ and CMh− animals. In general, anemia was associated with haemonchosis. Similar results were reported by Wagenfeld et al. who found no significant difference in hematocrit, Hb or RBC count when comparing laboratory data from 219 positive and 341 negative animals^52^.

With the exception of poikilocytosis, the other hematological parameters did not show any significant differences between CMh+ and CMh− animals. The degree of poikilocytosis was more frequent and more pronounced in CMh− animals, which could be explained by the fact that the highest degrees of poikilocytosis were observed in the severely anemic animals that revealed haemonchosis.

A particularly relevant finding was the occurrence of basophilic dots. These occurred in both CMh+ (4/13) and CMh− animals (4/22). There was no statistical difference in the occurrence of these dots between the two groups. This suggests, on the one hand, that light microscopy alone will not detect mycoplasma in every infected animal and, on the other hand, that there is a risk of false-positive results. Other authors also conclude that evaluation of a blood smear is less sensitive than performing a PCR^18,19^. Crosse et al. observed a *CMh−*infected alpaca for several months, and while DNA of the pathogen could be detected by PCR over a longer period, not all blood smears showed evidence of the pathogen^18^. This is consistent with the findings presented here: no pathogen was detected in the blood smear at the follow-up examination after oxatetracycline treatment, but the PCR still gave a positive result. The microscopical investigation of a blood smear alone is not sufficient for a definitive diagnosis. Although it can provide hints, a definitive diagnosis should be made by PCR testing^13,24^.

Observed erythrocyte inclusion bodies should be differentiated carefully. In addition to bacteria, they could be basophilic stippling, which are aggregates of ribosomes or polyribosomes^53^, or Howell-Jolly bodies, which are nuclear remnants^45^. While the dark spots in the case of basophilic stippling and Howell-Jolly bodies are found only intracellularly, hemotropic mycoplasmas can appear in, on, and between erythrocytes^7,12,54^. The importance of using fresh blood smears for light microscopic diagnosis has been reported. Due to the fact that mycoplasmas may otherwise detach from the erythrocytes^3,54^, fresh staining solutions should be used if possible, to avoid precipitates. As the blood samples from the animals in our study were all examined in the clinic's own laboratory, it can be assumed that examination of the samples was performed without unnecessary transport times and the trained laboratory personnel with special expertise in SAC hematology was able to distinguish different morphologies.

**Table 1:** Descriptive statistics on clinical and laboratory diagnostic data of hospitalized animals with negative (CMh−) and positive (CMh+) detection of *Candidatus* Mycoplasma haemolamae. References according to a) Whitehead^3^; b) Wagener et al.^55^; c) Lopez^56^; d) Dawson et al.^35^; e) Fowler and Zinkl^57^; f) Hajduk^42^.

Parameter	Unit	Reference	CMh− (negative)				CMh+ (positive)				*p* value
			n	Mean ± SD	Med	Min–Max	n	Mean ± SD	Med	Min–Max	
Age	days		22	1917.9 ± 1504.6	1515	206–5013	13	1611.2 ± 1185.6	1655	43–4056	0.7457
Hospitalization	days		19	14.7 ± 9.5	13	2–35	9	13.5 ± 7.1	15	1–25	0.7098
Body weight	kg		22	46.8 ± 20.0	48.3	10.4–102	12	51.2 ± 17.3	52	12.4–77.5	0.5249
Rectal temperature	°C	38.0–38.9 ^a)^	22	37.9 ± 1.1	38.2	35.0–39.6	12	38.5 ± 0.7	38.5	37.4–39.9	0.1431
Breathing rate	1/min	Adults: 15–30; Crias: 20–30 ^a)^	22	32.5 ± 10.2	34	16–48	10	33.7 ± 11.3	36	16–48	0.7765
Heart rate	1/min	Adults: 60–80; Crias: 70–100 ^a)^	21	89.0 ± 28.2	80	52–140	10	83.4 ± 33.3	73	54–148	0.7032
BCS		2,5–3,5 ^b)^	22	1.93 ± 0.88	1.5	1–4	12	2.63 ± 0.68	2.75	1.5–3.5	0.0145
FAMACHA©-Score		≤ 2 ^c)^	21	2.52 ± 1.72	2	1–5	9	1.55 ± 1.33	1	1–5	0.1378
GIN	1/g		22	184.9 ± 350.0	24.5	0–1239	13	28.7 ± 63.3	3	0–224	0.1185
WBC	×10^9^/L	7.1–18.6 ^d)^	22	15.2 ± 10.4	10.2	4.3–35.4	13	14.9 ± 6.7	14.4	6.3–27.6	0.5164
PCV	L/L	0.22–0.45 ^d)^	22	0.18 ± 0.09	0.19	0.04–0.33	13	0.21 ± 0.08	0.23	0.05–0.29	0.1989
Hb	g/L	102–193 ^d)^	22	73.2 ± 35.6	83	17–129	13	90.5 ± 34.3	101	17–128	0.1419
MCHC	g/L	420–490 ^d)^	22	421.0 ± 39.0	422.5	318–494	13	420.8 ± 30.0	425	340–457	0.9184
Normoblasts	1/100 WBC	0–3 ^d)^	22	14.0 ± 30.3	0	0–128	13	3.8 ± 9.1	0	0–27	0.3756
Lymphocytes	×10^9^/L	1.1–5.4 ^d)^	22	2.36 ± 1.82	1.95	0.24–7.43	13	2.40 ± 1.8	1.94	0.62–6.12	0.9049
Segmented neutrophils	×10^9^/L	3.5–11.7 ^d)^	22	10.17 ± 7.48	6.38	0.39–23.90	13	10.06 ± 5.86	8.29	1.56–22.94	0.5277
Band neutrophils	×10^9^/L	0 ^d)^	22	0.83 ± 1.00	0.4	0–4.31	13	1.31 ± 1.69	0.72	0–4.97	0.6324
Metamyelocytes	×10^9^/L	0	22	0.02 ± 0.05	0	0–0.16	13	0.04 ± 0.11	0	0–0.39	0.8664
Myelocytes	×10^9^/L	0	22	0 ± 0	0	0–0	13	0.01 ± 0.03	0	0–0.11	0.2143
Eosinophils	×10^9^/L	0.1–4.3 ^d)^	22	0.80 ± 1.10	0.32	0–3.78	13	0.66 ± 1.13	0.22	0–3.89	0.7711
Basophils	×10^9^/L	0–0.4 ^d)^	22	0.11 ± 0.11	0.08	0–0.37	13	0.05 ± 0.09	0	0–0.27	0.0785
Monocytes	×10^9^/L	0–1.0 ^d)^	22	0.41 ± 0.40	0.26	0–1.59	13	0.35 ± 0.24	0.39	0.07–0.79	1
Reticulocytes	% of RBC	0–2.4 ^e)^	13	120.7 ± 190.8	89	3–715	3	33.3 ± 56.0	2	0–98	0.1054
NLR		0.5–2.9 ^f)^	22	6.46 ± 5.83	3.87	0.46–23.13	13	7.21 ± 6.01	5.93	0.92–18.6	0.6946
LMR			21	8.93 ± 7.55	6.29	1.43–31	13	13.41 ± 24.72	6	2.29–79.00	0.9859
Anisocytosis			22	1.77 ± 0.87	1.5	1–3	13	1.38 ± 0.77	1	1–3	0.1607
Polychromasia			22	0.91 ± 1.02	1	0–3	13	1.00 ± 1.08	1	0–3	0.8261

Med: Median; Min: Minimum; Max: Maximum; BCS: Body condition score; GIN: Gastrointestinal nematodes; WBC: White blood count; PCV: packed cell volume; Hb: Hemoglobin; MCHC: Mean corpuscular haemoglobin concentration; NLR: Neutrophil-to-lymphocyte ratio; LMR: Lymphocyte-to-monocyte ratio. The *p* value indicates the results of the t-test or the Mann–Whitney U test.

**Table 2:** Contingency tables containing negative or positive PCR results for *Candidatus* Mycoplasma haemolamae and age, sex, clinical findings, and outcome.

		CMh− (negative)	CMh+ (positive)	sum	*p* value
Demographic data					
Age	Adult	19	12	31	1.000
	Cria	3	1	4	
	Sum	22	13	35	
Sex	Female	7	5	12	0.7260
	Male	15	8	23	
	sum	22	13	35	
Clinical findings					
Hypothermia	Yes	9	2	11	0.2525
	No	13	10	23	
	Sum	22	12	34	
Hyperthermia	Yes	1	2	3	0.2794
	No	21	10	31	
	Sum	22	12	34	
Bradypnea	Yes	1	0	1	1.000
	No	21	10	31	
	Sum	22	10	32	
Tachypnea	Yes	12	7	19	0.4673
	No	10	3	13	
	Sum	22	10	32	
Bradycardia	Yes	4	2	6	1.000
	No	17	8	25	
	Sum	21	10	31	
Tachycardia	Yes	10	2	12	0.2396
	No	11	8	19	
	Sum	21	10	31	
Emaciation	Yes	17	3	20	0.0048
	No	5	9	14	
	Sum	22	12	34	
Pale conjunctives	Yes	9	1	10	0.2035
	No	12	8	20	
	Sum	21	9	30	
Outcome					
Discharged	Yes	17	12	29	0.3771
	No	5	1	6	
	Sum	22	13	35	

The *p* value indicates the results of the Fisher exact test.

**Table 3:** Contingency tables containing negative or positive PCR results for *Candidatus* Mycoplasma haemolamae and laboratory findings.

		CMh− (negative)	CMh+ (positive)	sum	*p* value
Laboratory findings					
Anemia (PCV decreased)	Yes	14	4	18	0.0858
	No	8	9	17	
	Sum	22	13	35	
Hb decreased	Yes	16	7	23	0.2925
	No	6	6	12	
	Sum	22	13	35	
MCHC decreased	Yes	10	5	15	0.7372
	No	12	8	20	
	Sum	22	13	35	
Normoblasts	Yes	9	4	13	0.7212
	No	13	9	22	
	Sum	22	13	35	
Poikilocytosis	Yes	16	4	20	0.0322
	No	6	9	15	
	Sum	22	13	35	
Polychromasia	Yes	13	8	21	1.000
	No	9	5	14	
	Sum	22	13	35	
Basophilic dots	Yes	4	4	8	0.4327
	No	18	9	27	
	sum	22	13	35	
Howell-Jolly-bodies	Yes	2	0	2	0.5193
	No	20	13	33	
	Sum	22	13	35	
Cabot rings	Yes	5	1	6	0.3771
	No	17	12	29	
	Sum	22	13	35	
Leukopenia	Yes	4	2	6	1.000
	No	18	11	29	
	Sum	22	13	35	
Leukocytosis	Yes	7	2	9	0.4311
	No	15	11	26	
	Sum	22	13	35	
Lymphopenia	Yes	5	3	8	1.000
	No	17	10	27	
	Sum	22	13	35	
Lymphocytosis	Yes	2	1	3	1.000
	No	20	12	32	
	Sum	22	13	35	
Neutropenia	Yes	4	1	5	0.6300
	No	18	12	30	
	Sum	22	13	35	
Neutrophilia	Yes	8	3	11	0.4776
	No	14	10	24	
	Sum	22	13	35	
Eosinopenia	Yes	6	3	9	1.000
	No	16	10	26	
	Sum	22	13	35	
Eosinophilia	Yes	0	0	0	
	No	22	13	35	
	Sum	22	13	35	
Basophilia	Yes	0	0	0	
	No	22	13	35	
	Sum	22	13	35	
Monocytosis	Yes	2	0	2	0.5193
	No	20	13	33	
	Sum	22	13	35	
NLR increased	Yes	16	9	25	1.000
	No	6	4	10	
	Sum	22	13	35	
NLR decreased	Yes	1	0	1	1.000
	No	21	13	34	
	Sum	22	13	35	
Gastrointestinal nematodes	Yes	16	8	24	0.7077
	No	6	5	11	
	Sum	22	13	35	
*Eimeria macusaniensis*	Yes	2	0	2	0.5193
	No	20	13	33	
	Sum	22	13	35	

The *p* value indicates the results of the Fisher exact test.

**Table 4:** Overview of morphological findings in red blood cells in the blood smears of alpacas that were tested negative (CMh−) or positive (CMh+) for *Candidatus* Mycoplasma haemolamae.

	CMh− (negative)						CMh+ (positive)					
	Present	Grade					Present	Grade				
		0	1	2	3	4		0	1	2	3	4
Anisocytosis	22/22	0	11	5	6	0	13/13	0	10	1	2	0
Poikilocytosis	16/22	6	7	3	6	0	4/13	9	2	1	1	0
Polychromasia	13/22	9	9	1	3	0	8/13	5	5	1	2	0
Basophilic dots	4/22						4/13					
Howell-Jolly bodies	2/22						0/13					
Cabot rings	5/22						1/13					

As the animals presented to the clinic had often been ill for a long time, it was not known how long the *CMh* infections had been present at the time of presentation. This has to be considered a limitation of this retrospective study. It cannot be excluded that the animals had already passed through an acute phase associated with other clinical signs. The relatively small number of animals considered in this study represents a further limitation of the results.

## Conclusion

No clinical or hematological relevant differences between CMh+ and CMh− alpacas were found in our study. The clinical symptoms in the animals presented were caused by various comorbidities. The extent to which *CMh* influenced the course of the disease cannot be assessed due to the small number of animals. Even if *CMh* infects the erythrocytes, the pathogen does not appear to be a significant cause of anemia. *Haemonchus contortus* is instead considered to be the main reason for anemia in South American camelids. Diagnosis of *CMh* based solely on microscopic examination of a blood smear does not appear to be sufficient. Instead, PCR should be used to confirm or rule out infection. Although our study cannot exclude that *CMh* might lead to relevant clinical disease, antibiotic treatment based only on a positive test result should be critically questioned in the light of our results.

## Data Availability

The original contributions presented in the study are included in the article. Further inquiries can be directed to the corresponding author/s.

## Abbreviations

BCS: Body condition score
CMh: *Candidatus* Mycoplasma haemolamae
CMh+: *Candidatus* Mycoplasma haemolamae positive animals
CMh−: *Candidatus* Mycoplasma haemolamae negative animals
GIN: Gastrointestinal nematodes
Hb: Hemoglobin
LMR: Lymphocyte-to-monocyte ratio
Max: Maximum
MCHC: Mean corpuscular hemoglobin concentration
Med: Median
Min: Minimum
NLR: Neutrophil-to-lymphocyte ratio
PCR: Polymerase chain reaction
PCV: Packed cell volume
SAC: South American camelid
RBC: Red blood count
WBC: White blood count

## References

[1] Wagner, H., Ulrich, L., Leisen, A. & Wehrend, A. Population structure of South American camelids in Germany. *Tierarztl. Prax. Ausg. G Grosstiere Nutztiere.* **50**, 237–249. 10.1055/a-1899-5786 (2022). 36067758 10.1055/a-1899-5786

[2] Neubert, S. *South American camelids in Germany – Investigations into Husbandry, Management Measures and Common Diseases* (University of Veterinary Medicine Hannover, 2022).

[3] Whitehead, C. Diseases in camelids 1. Common presentations. *In. Pract.* **35**, 317–324. 10.1136/inp.f3641 (2013). 10.1136/inp.f3641

[4] Wagener, M. G., Neubert, S., Punsmann, T. M., Wiegand, S. B. & Ganter, M. Relationships between Body Condition Score (BCS), FAMACHA©-Score and Haematological Parameters in Alpacas (*Vicugna pacos*), and Llamas (*Lama glama*) Presented at the Veterinary Clinic. *Animals.* **11**, 2517. 10.3390/ani11092517 (2021). 34573483 10.3390/ani11092517 PMC8469494

[5] Wagener, M. G., Puff, C., Stöter, M., Schwennen, C. & Ganter, M. Regenerative anaemia in alpacas (*Vicugna pacos*) can lead to a wrong diagnosis of leucocytosis. *Vet. Rec. Case Rep.* **8**, e001257. 10.1136/vetreccr-2020-001257 (2020). 10.1136/vetreccr-2020-001257

[6] Edwards, E. E., Garner, B. C., Williamson, L. H., Storey, B. E. & Sakamoto, K. Pathology of *Haemonchus contortus* in New World camelids in the southeastern United States: A retrospective review. *J. Vet. Diagn. Invest.* **28**, 105–109. 10.1177/1040638716628587 (2016). 26965230 10.1177/1040638716628587

[7] Messick, J. B. Hemotrophic mycoplasmas (hemoplasmas): A review and new insights into pathogenic potential. *Vet. Clin. Pathol.* **33**, 2–13. 10.1111/j.1939-165X.2004.tb00342.x (2004). 15048620 10.1111/j.1939-165X.2004.tb00342.x

[8] Vap, L. & Bohn, A. A. Hematology of camelids. *Vet. Clin. North. Am. Exot. Anim. Pract.* **18**, 41–49. 10.1016/j.cvex.2014.09.010 (2015). 25421025 10.1016/j.cvex.2014.09.010

[9] Tornquist, S., Boeder, L., Lubbers, S. & Cebra, C. Investigation of Mycoplasma haemolamae infection in crias born to infected dams. *Vet. Rec.* **168**, 380–380. 10.1136/vr.c6735 (2011). 10.1136/vr.c6735 21498269

[10] Almy, F. S., Ladd, S. M., Sponenberg, D. P., Crisman, M. V. & Messick, J. B. Mycoplasma haemolamae infection in a 4-day-old cria: Support for in utero transmission by use of a polymerase chain reaction assay. *Can. Vet. J.* **47**, 229–233 (2006). 16604978 PMC1371050

[11] Pentecost, R. L. *et al.* Vertical transmission of Mycoplasma haemolamae in alpacas (*Vicugna pacos*). *Small. Rumin. Res.* **106**, 181–188. 10.1016/j.smallrumres.2012.02.021 (2012). 10.1016/j.smallrumres.2012.02.021

[12] McLaughlin, B. G. *et al.* An Eperythrozoon-like parasite in llamas. *J. Am. Vet. Med. Assoc.* **197**, 1170–1175 (1990). 2254144 10.2460/javma.1990.197.09.1170

[13] Tornquist, S., Boeder, L., Parker, J., Cebra, C. & Messick, J. Use of a polymerase chain reaction assay to study the carrier state in infection with camelid Mycoplasma haemolama, formerly *Eperythrozoon* spp. infecting camelids. *Vet. Clin. Pathol.* **31**, 153–154 (2002).

[14] Tornquist, S. J., Boeder, L., Rios-Phillips, C. & Alarcon, V. Prevalence of Mycoplasma haemolamae infection in Peruvian and Chilean llamas and alpacas. *J. Vet. Diagn. Invest.* **22**, 766–769. 10.1177/104063871002200520 (2010). 20807939 10.1177/104063871002200520

[15] Kaufmann, C., Meli, M., Hofmann-Lehmann, R., Riond, B. & Zanolari, P. Epidemiology of “Candidatus Mycoplasma haemolamae” infection in South American camelids in Central Europe. *J. Camelid Sci.* **4**, 23–29 (2011).

[16] Kaufmann, C., Meli, M. L., Hofmann-Lehmann, R. & Zanolari, P. First detection of “Candidatus Mycoplasma haemolamae” in South American Camelids of Switzerland and evaluation of prevalence. *Berl. Munch. Tierarztl. Wochenschr.* **123**, 477–481. 10.2376/0005-9366-123-477 (2010). 21141277 10.2376/0005-9366-123-477

[17] Franz, S. *et al.* “Candidatus Mycoplasma haemolamae” infections in clinically asymptomatic Austrian South American Camelids. *Berl. Munch. Tierarztl. Wochenschr.* **129**, 318–322. 10.2376/0005-9366-15082 (2016). 27529994 10.2376/0005-9366-15082

[18] Crosse, P. *et al.* First detection of ‘Candidatus Mycoplasma haemolamae’ infection in alpacas in England. *Vet. Rec.* **171**, 71–75. 10.1136/vr.100611 (2012). 22781345 10.1136/vr.100611

[19] Dittmer, K., Hinkson, J., Dwyer, C., Adlington, B. & van Andel, M. Prevalence of Candidatus Mycoplasma haemolamae, bovine viral diarrhoea virus, and gastrointestinal parasitism in a sample of adult New Zealand alpaca (*Vicugna pacos*). *N. Z. Vet. J.* **66**, 9–15. 10.1080/00480169.2017.1369912 (2018). 28826356 10.1080/00480169.2017.1369912

[20] Sala, G., Ratti, G., Ferrulli, V., Scavone, D., Stranieri, A., Giordano, A., Boccardo, A., Pravettoni, D. & Lauzi, S. First detection of “Candidatus Mycoplasma haemolamae” in alpaca (*Vicugna pacos*) in Italy. In *Proceedings of the World Buiatric Congress*, Madrid, Spain, Sept 4th to 8th, The World Association for Buiatrics (WAB), BC-04 (2022).

[21] Leiskamo, K. *Mycoplasma haemolamae suomalaisilla alpakoilla ja laamoilla* (University of Helsinki, 2022).

[22] Viesselmann, L. C. *et al.* Mycoplasma haemolamae and intestinal parasite relationships with erythrocyte variables in clinically healthy alpacas and llamas. *J. Vet. Int. Med.* **33**, 2336–2342. 10.1111/jvim.15596 (2019). 10.1111/jvim.15596 PMC6766537 31454105

[23] Luethy, D., Stefanovski, D., Salber, R. & Sweeney, R. Prediction of packed cell volume after whole blood transfusion in small ruminants and South American Camelids: 80 Cases (2006–2016). *J. Vet. Int. Med.* **31**, 1900–1904. 10.1111/jvim.14844 (2017). 10.1111/jvim.14844 PMC5697174 28961345

[24] Meli, M. L. *et al.* Development and application of a real-time TaqMan® qPCR assay for detection and quantification of ‘Candidatus Mycoplasma haemolamae’ in South American camelids. *Vet. Microbiol.* **146**, 290–294. 10.1016/j.vetmic.2010.05.029 (2010). 21095509 10.1016/j.vetmic.2010.05.029

[25] Tornquist, S. J., Boeder, L. J., Cebra, C. K. & Messick, J. Use of a polymerase chain reaction assay to study response to oxytetracycline treatment in experimental Candidatus Mycoplasma haemolamae infection in alpacas. *Am. J. Vet. Res.* **70**, 1102–1107. 10.2460/ajvr.70.9.1102 (2009). 19719425 10.2460/ajvr.70.9.1102

[26] Rüfli, I. *et al.* Causes of abortions in south American camelids in Switzerland—cases and questionnaire. *Animals.* **11**, 1956. 10.3390/ani11071956 (2021). 34208975 10.3390/ani11071956 PMC8300385

[27] Hart, J. C., Burton, A. J., Pinn, T. L., Fubini, S. L. & Dawson, D. R. Spiral colon impaction in juvenile alpacas: 12 cases (2006–2010). *J. Am. Vet. Med. Assoc.* **242**, 1419–1424. 10.2460/javma.242.10.1419 (2013). 23634688 10.2460/javma.242.10.1419

[28] O’Conor Dowd, M. C. *Diseases of New World Camelids* (University of Minnesota, 2014).

[29] Clarke, L. L. & Breuer, R. M. Postmortem diagnoses in South American camelids and factors influencing diagnostic rate in the Upper Midwest USA, 2009–2019. *J. Vet. Diagn. Invest.* **34**, 727–732. 10.1177/10406387221091733 (2022). 35394374 10.1177/10406387221091733 PMC9266521

[30] Twomey, D., Wu, G., Nicholson, R., Watson, E. & Foster, A. Review of laboratory submissions from New World camelids in England and Wales (2000–2011). *Vet. J.* **200**, 51–59. 10.1016/j.tvjl.2014.01.021 (2014). 24721312 10.1016/j.tvjl.2014.01.021

[31] Björklund, C. *Diseases and Causes of Death among Camelids in Sweden* (Swedish University of Agricultural Sciences, 2014).

[32] Theuß, T., Goerigk, D., Rasenberger, S., Starke, A. & Schoon, H.-A. Pathology of South American Camelids: A retrospective study of necropsies at the Institute of Veterinary Pathology, University of Leipzig, Germany. *Tierarztl. Prax. Ausg. G Grosstiere Nutztiere.* **42**, 278–288. 10.1055/s-0038-1623237 (2014). 25327150 10.1055/s-0038-1623237

[33] Wagener, M. G., Grossmann, T., Stöter, M. & Ganter, M. Hematological diagnostics in llamas and alpacas. *Prakt. Tierarzt.* **99**, 481–493. 10.2376/0032-681X-18-10 (2018). 10.2376/0032-681X-18-10

[34] Bauer, B. U. *et al.* Anaplasma phagocytophilum and Anaplasma ovis—Emerging Pathogens in the German Sheep Population. *Pathogens.* **10**, 1298. 10.3390/pathogens10101298 (2012). 10.3390/pathogens10101298 PMC8537415 34684247

[35] Dawson, D. R., DeFrancisco, R. J. & Stokol, T. Reference intervals for hematologic and coagulation tests in adult alpacas (*Vicugna pacos*). *Vet. Clin. Pathol.* **40**, 504–512. 10.1111/j.1939-165X.2011.00359.x (2011). 22092869 10.1111/j.1939-165X.2011.00359.x

[36] Wittek, T. & Franz, S. Anämie. In *Praxishandbuch Neuweltkamele Ein Leitfaden zur Diagnostik, Therapie Und Prophylaxe Bei Lamas Und Alpakas* (eds Wittek, T. & Franz, S.) 79–83 (Schlütersche, 2021).

[37] Wagener, M. G. & Ganter, M. Body Condition Scoring in South American camelids. *Prakt. Tierarzt.* **101**, 684–696. 10.2376/0032-681X-2020 (2020). 10.2376/0032-681X-2020

[38] Wagener, M., Meyer zu Westerhausen, M., Neubert, S. & Ganter, M. Identification of anaemic alpacas and llamas using the FAMACHA© score. *Prakt. Tierarzt.* **103**, 620–629. 10.2376/0032-681X-2227 (2022). 10.2376/0032-681X-2227

[39] Roden, E. *Retrospective Analysis of Small Ruminant Fecal Exams (2007–2016) and Identification of Haemonchus contortus* (University of Veterinary Medicine Hannover, 2022).

[40] Neubert, S. *et al.* Gastric ulcers in alpacas—clinical, laboratory, and pathological findings. *Front. Vet. Sci.* **9**, 877257. 10.3389/fvets.2022.877257 (2022). 35664847 10.3389/fvets.2022.877257 PMC9159277

[41] Bath, G. F., Malan, F. & Van Wyk, J. The “FAMACHA” ovine anaemia guide to assist with the control of haemonchosis. In *Proceedings of the 7th Annual Congress of the Livestock Health and Production Group of the South African Veterinary Association*, Port Elizabeth, South Africa, 5–7 June 1996, 5 (1996).

[42] Hajduk, P. Haematological reference values for alpacas. *Aust. Vet. J.* **69**, 89–90. 10.1111/j.1751-0813.1992.tb15558.x (1992). 1605791 10.1111/j.1751-0813.1992.tb15558.x

[43] Roche, J. R. *et al.* Invited review: Body condition score and its association with dairy cow productivity, health, and welfare. *J. Dairy. Sci.* **92**, 5769–5801. 10.3168/jds.2009-2431 (2009). 19923585 10.3168/jds.2009-2431

[44] Fisher, D. & Zinkl, J. Eperythrozoonosis in a one-day-old llama. *Vet. Clin. Pathol.* **25**, 93–94. 10.1111/j.1939-165x.1996.tb01002.x (1996). 12660969 10.1111/j.1939-165x.1996.tb01002.x

[45] Wagener, M. G., Marahrens, H. & Ganter, M. Anaemia in South American camelids—An overview of clinical and laboratory diagnostics. *Vet. Res. Commun.* **48**, 633-647. 10.1007/s11259-023-10274-z (2024). 38049672 10.1007/s11259-023-10274-z PMC10998796

[46] Colditz, I., Le Jambre, L. & Hosse, R. Use of lectin binding characteristics to identify gastrointestinal parasite eggs in faeces. *Vet. Parasitol.* **105**, 219–227. 10.1016/S0304-4017(02)00013-4 (2002). 11934462 10.1016/S0304-4017(02)00013-4

[47] Van Wyk, J. A. & Bath, G. F. The FAMACHA system for managing haemonchosis in sheep and goats by clinically identifying individual animals for treatment. *Vet. Res.* **33**, 509–529. 10.1051/vetres:2002036 (2002). 12387487 10.1051/vetres:2002036

[48] Storey, B. E. *et al.* Validation of the FAMACHA© system in South American camelids. *Vet. Parasitol.* **243**, 85–91. 10.1016/j.vetpar.2017.06.004 (2017). 28807317 10.1016/j.vetpar.2017.06.004

[49] Galvan, N., Middleton, J. R., Nagy, D. W., Schultz, L. G. & Schaeffer, J. W. Anthelmintic resistance in a herd of alpacas (*Vicugna pacos*). *Can. Vet. J.* **53**, 1310–1313 (2012). 23729829 PMC3500124

[50] Morin, D., Garry, F., Weiser, M., Fettman, M. & Johnson, L. Hematologic features of iron deficiency anemia in llamas. *Vet. Pathol.* **29**, 400–404. 10.1177/030098589202900505 (1992). 1413407 10.1177/030098589202900505

[51] Jones, M. L. & Allison, R. W. Evaluation of the ruminant complete blood cell count. *Vet. Clin. North. Am. Exot. Anim. Pract.* **23**, 377–402. 10.1016/j.cvfa.2007.07.002 (2007). 10.1016/j.cvfa.2007.07.002 17920454

[52] Wagenfeld, S., Golob, A., Müller, E. & Baur-Kaufhold, A. Comparison of the red blood count of South American Camelids that tested PCR positive for Candidatus Mycoplasma haemolamae with those that tested PCR negative. In *Proceedings of the South America Camelid Congress Vienna 2023*, Austria, 8–9 Dec 2023, 21–22 (2023).

[53] Harvey, J. W. The erythrocyte: Physiology, metabolism, and biochemical disorders. In *Clinical Biochemistry of Domestic Animals* 5th edn (eds Kaneko, J. J. *et al.*) 157–203 (Elsevier, 1997).

[54] Tornquist, S. J. Clinical pathology of llamas and alpacas. *Vet. Clin. North. Am. Exot. Anim. Pract.* **25**, 311–322. 10.1016/j.cvfa.2009.03.004 (2009). 10.1016/j.cvfa.2009.03.004 19460642

[55] Wagener, M. G., Ganter, M. & Leonhard-Marek, S. Body condition scoring in alpacas (*Vicugna pacos*) and llamas (*Lama **glama*)—A scoping review. *Vet. Res. Commun. ***48**, 665-684. 10.1007/s11259-023-10275-y (2024). 38133845 10.1007/s11259-023-10275-y PMC10998785

[56] Lopez, B. Approach to veterinary management of adult camelids. *In. Pract.* **43**, 329–337. 10.1002/inpr.81 (2021). 10.1002/inpr.81

[57] Fowler, M. & Zinkl, J. Reference ranges for hematologic and serum biochemical values in llamas (*Lama glama*). *Am. J. Vet. Res.* **50**, 2049–2053 (1989). 2610431

